# Hypertrophy and Dilatation of the Heart during Scarlet-Fever Nephritis

**Published:** 1882-02

**Authors:** 


### Hypertrophy and Dilatation of the Heart during Scarlet-Fever Nephritis.

Dr. Oscar Silbermann in an elaborate article on this subject speaks of the comparatively few observations which have been made in this direction up to the time of Friedlander's investigations during the year 1881. This author found, on examining a large number of cases of nephritis occurring during attacks of scarlet fever, decided hypertrophy of the heart combined with dilatation, in some cases both sides of the heart being equally affected, but usually only the left side; he also made a careful comparison of the weights of hearts in healthy children with those who had died of scarlet-fever nephritis. In only a few cases was there found a partial fatty degeneration of the muscular fibres; the endocardium, pericardium, and blood vessels were normal. There was no doubt according to these observations that the cardiac affection was related to the postscarlatinal nephritis and not to the scarlet-fever process itself, as the hypertrophy was never found where the children died in the early weeks of the disease. Silbermann draws attention to the short period which intervened between the first appearance of the nephritis and the consecutive heart hypertrophy, in many of the cases the time being not much longer than a week, and he refers to the physiological observations of Beneke and Gerhardt, which showed that the heart between the third and eighth year is relatively larger than in adult life, and that at this age there exists a physiological hypertrophy of the left ventricle, caused by a continuance of the aortic narrowing in the neighborhood of the ductus Botalli, and Silbermann thinks that this tendency to physiological hypertrophy between the ages of three and eight may account for the speedy dilatation and hypertrophy following the acute nephritis of scarlet fever, the cases in which it occurred being respectively four, five, six, three and a half, and six years old. —*Boston Medical and Surgical Journal.*

